# Supplementary material for: ABHD4-dependent developmental anoikis safeguards the embryonic brain
Source: Nat Commun. 2020 Aug 31;11:4363. doi: 10.1038/s41467-020-18175-4 (PMC7459116; doi:10.1038/s41467-020-18175-4)
Supplement: Supplementary file 1 — Supplementary Information [file 41467_2020_18175_MOESM1_ESM.pdf]

Supplementary Information

ABHD4-dependent developmental anoikis safeguards the embryonic brain

László et al.

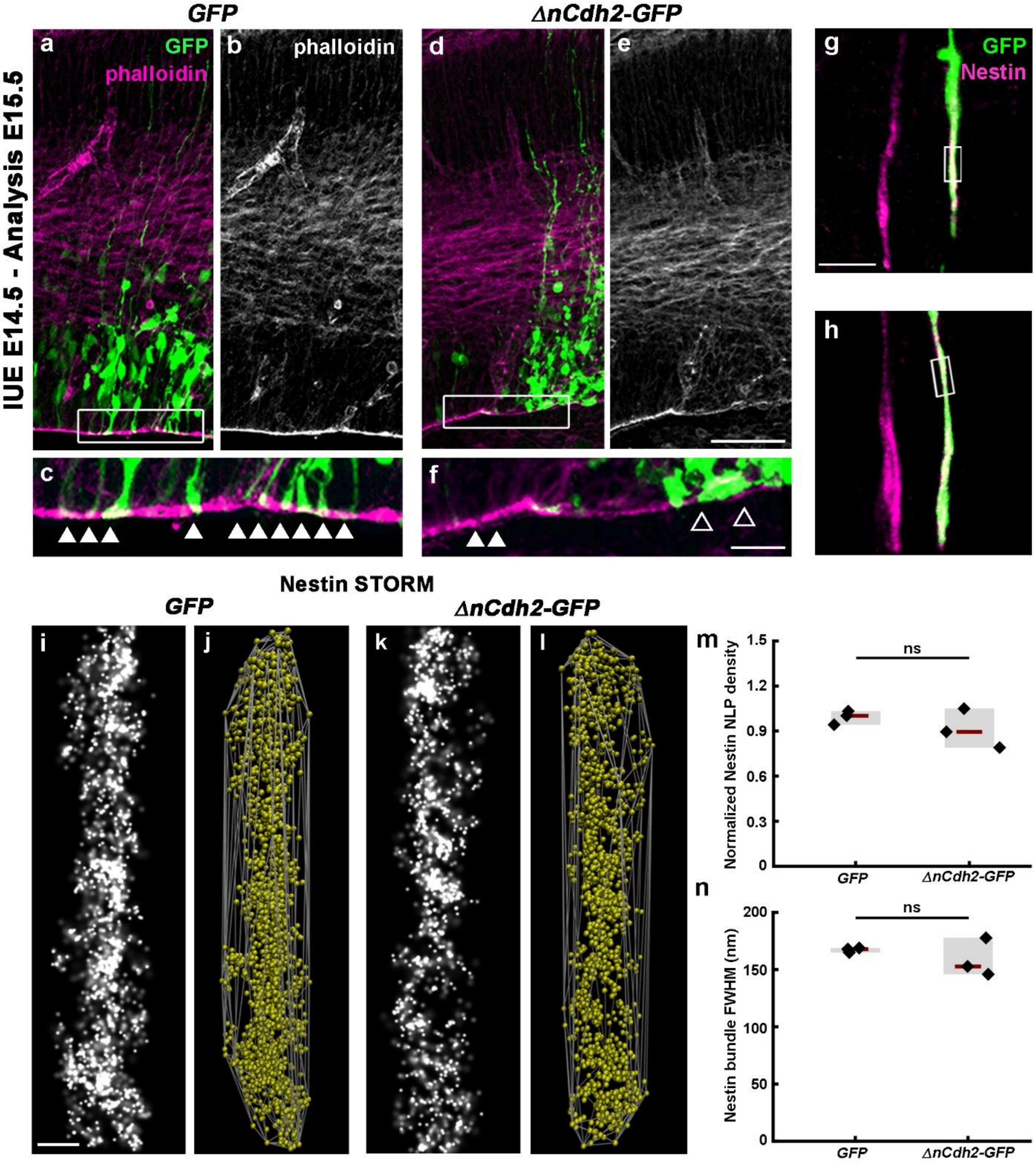

Supplementary Figure 1.

Supplementary Fig. 1. Disconnection of apical adherens junction does not affect the radial processes of RGPCs. a-f Low- (**a,b,d,e**) and high (**c,f**)-power confocal images of the embryonic neocortex stained with the F-actin marker phalloidin that visualizes adherens junctions. MZ, marginal zone; CP, cortical plate; IZ, intermediate zone; SVZ, subventricular zone; VZ, ventricular zone. Note that *ΔnCdh2-GFP*-electroporation (IUE) specifically dismantles the adherens junction belt around the affected apical endfeet of RGPCs (**f**), whereas adjacent areas remain intact (white arrowheads), as in control experiments (**c**). **g-l** The nanoarchitecture of radial glia processes remains intact. Confocal images of nestin-immunostaining in *GFP*- (**g**) and *ΔnCdh2-GFP*-electroporated (**h**) radial processes. STORM super-resolution images of nestin filaments (**i,k**), and 3D-convex hulls fitted on the nestin localization points to visualize the intermediate filament architecture (**j,l**). **m-n** Statistical analysis of the density of the number of localization points (NLP) representing nestin (**m**) and the full-width-at-half-maximum of nestin filament bundles (two-sided Mann-Whitney U test, ns = not significant, $P = 0.7$ (**m**) and $P = 0.55$ (**n**); $n = 3$ animals in all cases). Graphs show box-and-whisker plots (including minima, maxima and median values, and lower and upper quartiles) with values representing averages per animals. Scale bars: **a-f**: 50 µm; **g-h**: 5 µm; **i-l**: 200 nm. Source data are provided as a Source Data file.

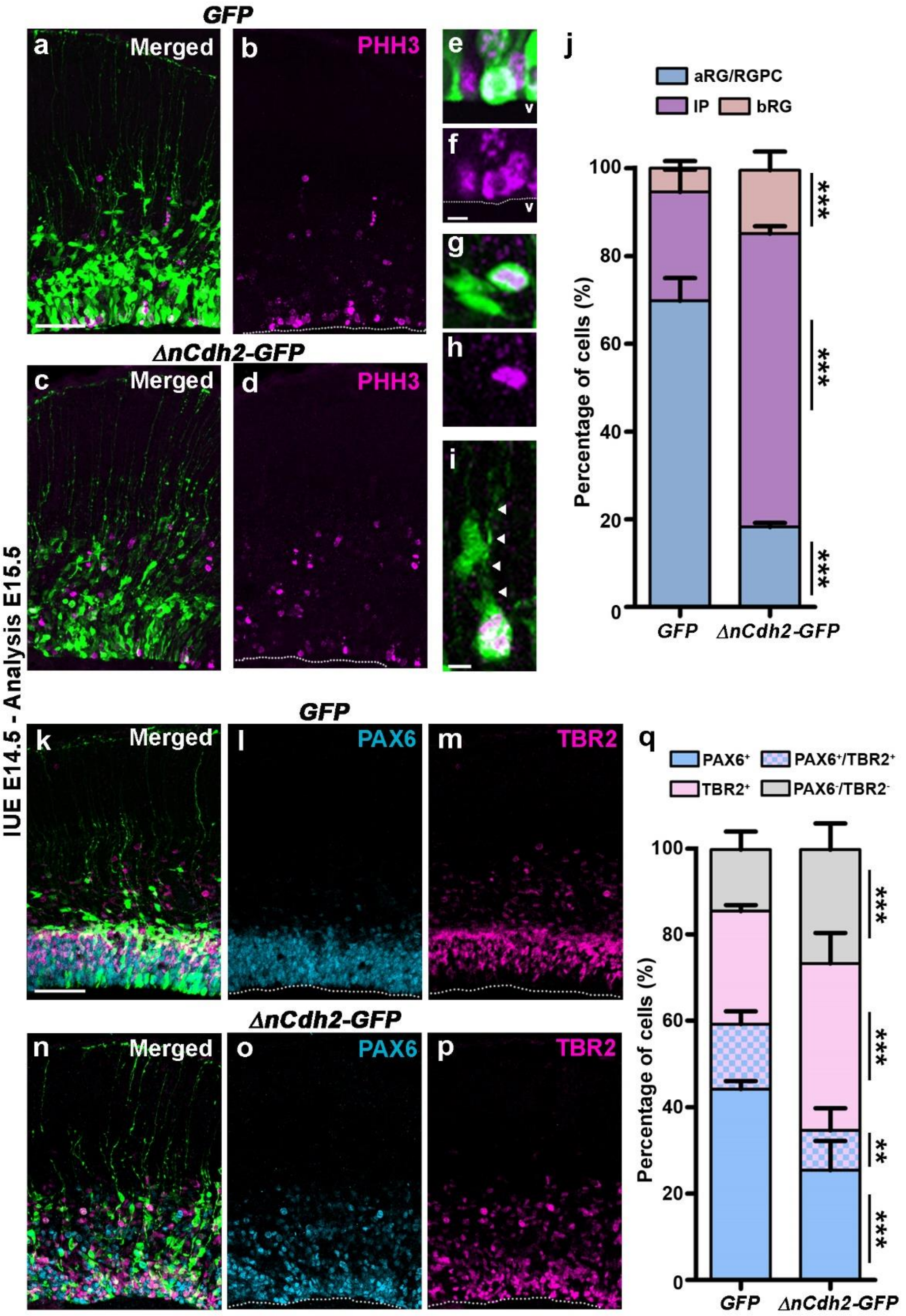

Supplementary Figure 2.

Supplementary Fig. 2. Elimination of N-cadherin-based apical adherens junctions affects differentiation. a-d Selective adherens junction disruption causes cell type changes and the disappearance of PHH3-positive dividing cells from the ventricular surface (**c-d**) compared control condition (**a-b**). **e-i** Distinct dividing cell types in the embryonic cortex: apical radial glia cell (aRG) or RGPC (**e-f**) at the apical surface, intermediate progenitor cell (IP, **g-h**) in the subventricular zone and basal radial glia cell (bRG, **i**) with basal projection. **j** Statistical analysis of the three distinct morphological cell type based on PHH3-immunostaining (two-sided Mann-Whitney U test, ***$P < 0.0001$; n = 19 sections from $n = 3$ animals in both treatment). **k-p** *ΔnCdh2-GFP*-electroporation (IUE), but not control *GFP* (**l-m**) promotes the disposition of the PAX6- (**o**) and TBR2-positive cells (**p**). Quantification of cell fate changes after *GFP*- and *ΔnCdh2-GFP*-electroporation based on cell fate markers (two-sided Mann-Whitney U test, ***$P < 0.0001$; **$P = 0.003$; n = 32 section in *GFP*- and $n = 34$ in *ΔnCdh2-GFP*-electroporated samples from $n = 4$ animals in both treatment. Graphs show bar plots with median ± interquartile range. Scale bars: **a-d, k-p**: 50 µm; **e-i**: 5 µm. Source data are provided as a Source Data file.

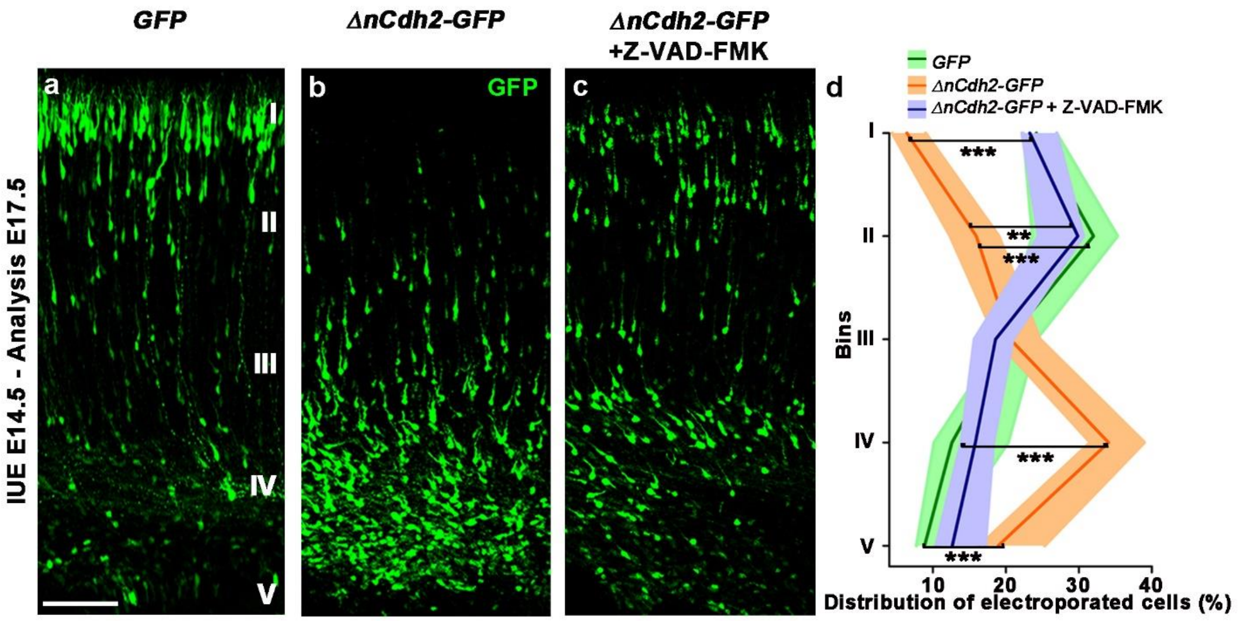

Supplementary Fig. 3. Loss of adherens junctions causes a migration defect. a,b In contrast to migration to the cortical plate under control conditions (**a**), abnormally delaminated cells are concentrated within the subventricular zone (**b**). **c** Notably, preclusion of cell death by the pan-caspase-inhibitor Z-VAD-FMK treatment restores radial migration. **d** Quantification of electroporated cells in five equally-sized bins (Roman numerals) (Kruskal-Wallis test with post hoc Dunn's test, 1st,4th bin (*GFP* vs *ΔnCdh2-GFP*, *ΔnCdh2-GFP* vs *ΔnCdh2-GFP* + Z-VAD-FMK): ***$P < 0.0001$; 5th bin (*GFP* vs *ΔnCdh2-GFP, ΔnCdh2-GFP* vs *ΔnCdh2-GFP* + Z-VAD-FMK): ***$P = 0.0001$; 2nd bin (*GFP* vs *ΔnCdh2-GFP): ****$P = 0.0002$; 2nd bin (*ΔnCdh2-GFP* vs *ΔnCdh2-GFP* + Z-VAD-FMK *): ***$P = 0.0031$; $n = 10$ sections from $n = 3$ animals per *GFP* treatment; $n = 12$ sections from $n = 3$ animals per *ΔnCdh2-GFP* treatment; $n = 14$ sections from $n = 3$ animals per *ΔnCdh2-GFP* + Z-VAD-FMK treatment). Data are shown as median (line) and interquartile range (transparent band in the same color). IUE, *in utero* electroporation. Scale bars: **a-c**: 50 µm. Source data are provided as a Source Data file.

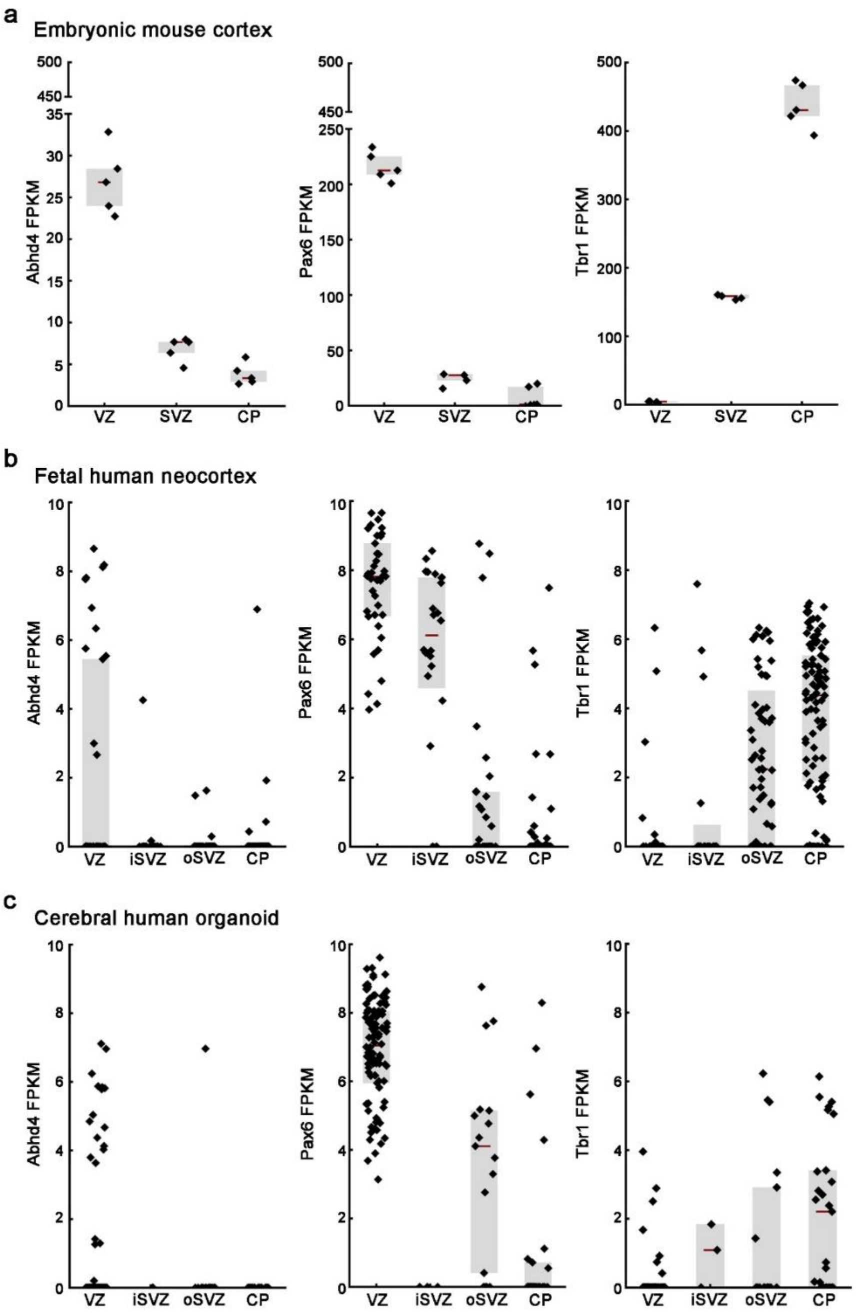

Supplementary Fig. 4. Single-cell RNA-sequence data demonstrates that *Abhd4* mRNA expression is restricted to the ventricular zone. a-c *In silico* data analysis of *Abhd4* mRNA distribution in distinct cortical zones from embryonic mouse cortex based on Fietz et al. 2012[27] (**a**), and from the human fetal cortex (**b**) and cerebral organoids (**c**) based on Camp et al. 2015[26]. All three datasets show high expression in the ventricular zone mirroring the expression of the RGPC marker, *Pax6*. In contrast, *Abhd4* displays complementary expression profile to *Tbr1*, cortical plate marker.

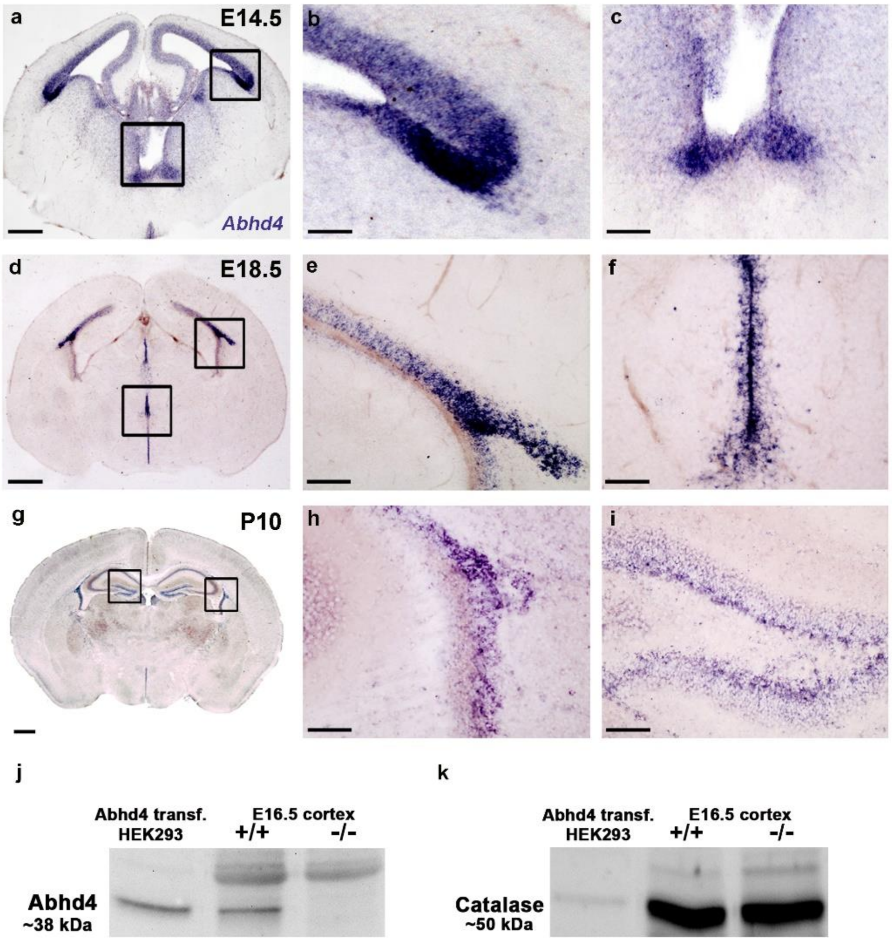

Supplementary Fig. 5. Spatiotemporally uniform *Abhd4* expression pattern in the germinative niches of the developing brain. a-i Coronal sections through the forebrain during prenatal and early postnatal development. Low-power light micrographs taken at embryonic days E14.5 (**a**) and E18.5 (**d**) and postnatal days P10 (**g**) highlight the consistently high expression of *Abhd4* mRNA in the proliferative zones throughout development. High-power light micrographs obtained from the lateral (**b,e**) and the third ventricle (**c,f**) and from the subventricular (**h**) and subgranular layers (**i**) demonstrate that *Abhd4* mRNA levels are under the detection threshold outside of the germinative niches.**j** Western blots verify ABHD4 protein in control *Abhd4-GFP*-transfected HEK293 cells and in the developing neocortex of wild-type (+/+), but not *Abhd4*-knockout mice at E16.5. **k** Immunoblotting for catalase is used as a loading control. Scale bars: **a**: 200 µm; **d, g**: 500 µm; **b, c, e, f, h, i**: 50 µm. Source data are provided as a Source Data file.

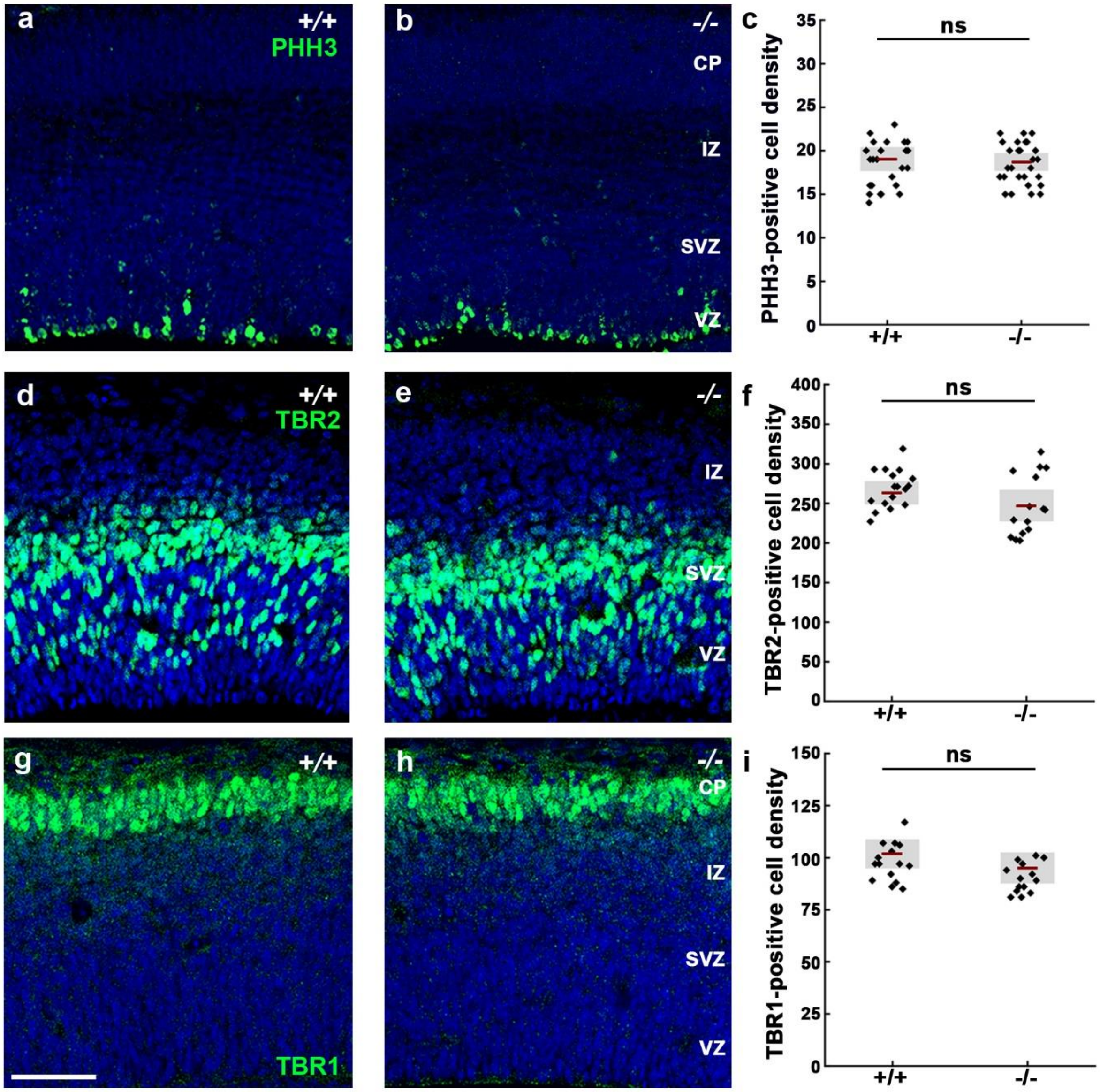

Supplementary Figure 6.

Supplementary Fig. 6. ABHD4 is not required for proliferation, differentiation and lamination in the developing neocortex. a,b Phospho-histone H3 (PHH3)-immunostaining visualizes cell proliferation during the M phase of the cell cycle at the ventricular border. **c,** Quantification of PHH3-positive cell density (two-sided Student's unpaired t-test, P = 0.688; n = 26 sections from n = 3 animals per wild-type (+/+) mice, n = 30 sections from n = 3 animals per *Abhd4*-knockout (-/-) mice). **d,e** High density of T-box brain protein 2 (TBR2)-positive intermediate progenitor cells in the subventricular zone. **f** Quantification of TBR2-positive cell density (two-sided Student's unpaired t-test, P = 0.194; n = 18 sections from n = 3 animals per wild-type (+/+) mice, n = 15 sections from n = 3 animals per *Abhd4*-knockout (-/-) mice). **g,h** Distribution of TBR1-positive postmitotic neurons in the cortical plate. **i** Quantification of TBR1-positive cell density (two-sided Mann-Whitney U test, P = 0.074; n = 17 sections from n = 3 animals per wild-type (+/+) mice, n = 16 sections from n = 3 animals per *Abhd4*-knockout (-/-) mice). MZ: marginal zone, CP: cortical plate, IZ: intermediate zone, SVZ: subventricular zone, VZ: ventricular zone. Graphs show box-and-whisker plots (including minima, maxima and mean values with 2 × standard error, except **(i)** where median and lower, upper quartiles are presented) with single values. Scale bar: **a-h:** 50 µm. Source data are provided as a Source Data file.

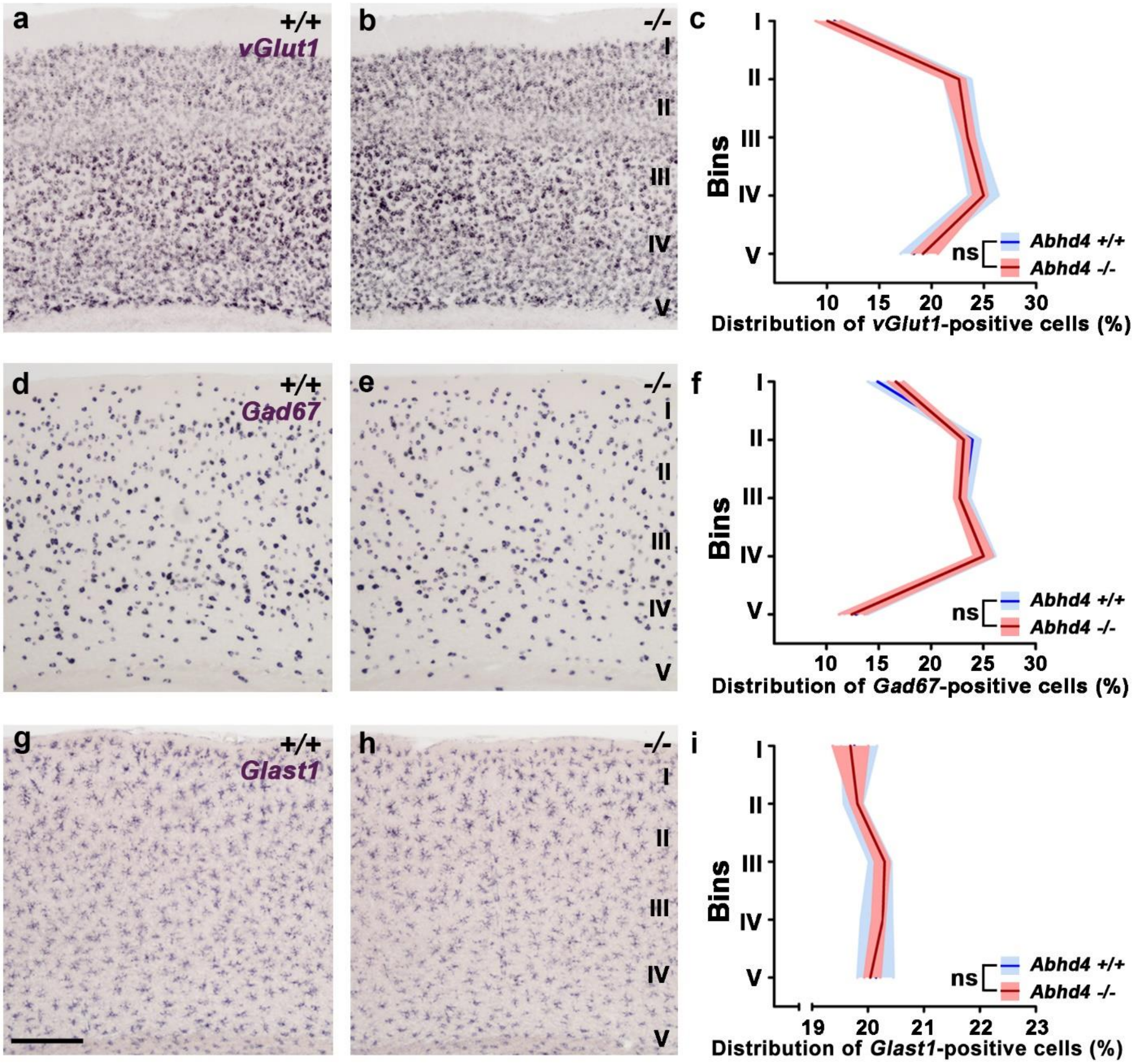

Supplementary Figure 7.

Supplementary Fig. 7. ABHD4 is not necessary for the differentiation and laminar distribution of adult cortical cell types. a,b Distribution of vGlut1-positive excitatory cells in coronal sections of the adult neocortex of wild-type (+/+) and *Abhd4*-knockout (-/-) mice. **c** Laminar density of *in situ* hybridization signal for *Slc17a7* (encoding the VGLUT1 protein). **d,e** Distribution of *Gad67*-positive inhibitory cells in coronal sections of the adult neocortex of wild-type (+/+) and *Abhd4*-knockout (-/-) mice. **f** Laminar density of *in situ* hybridization signal for *Gad1* (encoding the GAD67 protein). **g,h** Distribution of *Glast1*-positive astrocytes in coronal sections of the adult neocortex of wild-type (+/+) and *Abhd4*-knockout (-/-) mice. **i** Laminar density of *in situ* hybridization signal for *Slc1a3* (encoding the GLAST1 protein). Density measurements were performed in five equally-sized bins (Roman numerals) (two-sided Mann-Whitney U test , $P > 0.05$ in all bins of all experiments, see accompanying Supplementary data 1. for exact values; $n = 22$ sections from $n = 3$ animals per wild-type (+/+) mice, $n = 26$ sections from $n = 3$ animals per *Abhd4*-knockout (-/-) mice in case of vGlut1; $n = 23$ sections from $n = 3$ animals per wild-type (+/+) mice, $n = 24$ sections from $n = 3$ animals per *Abhd4*-knockout (-/-) mice in case of Gad67 and Glast1). Data are shown as median (line) and interquartile range (transparent band in the same color). Scale bar: **a-h:** 50 µm. Source data are provided as a Source Data file.

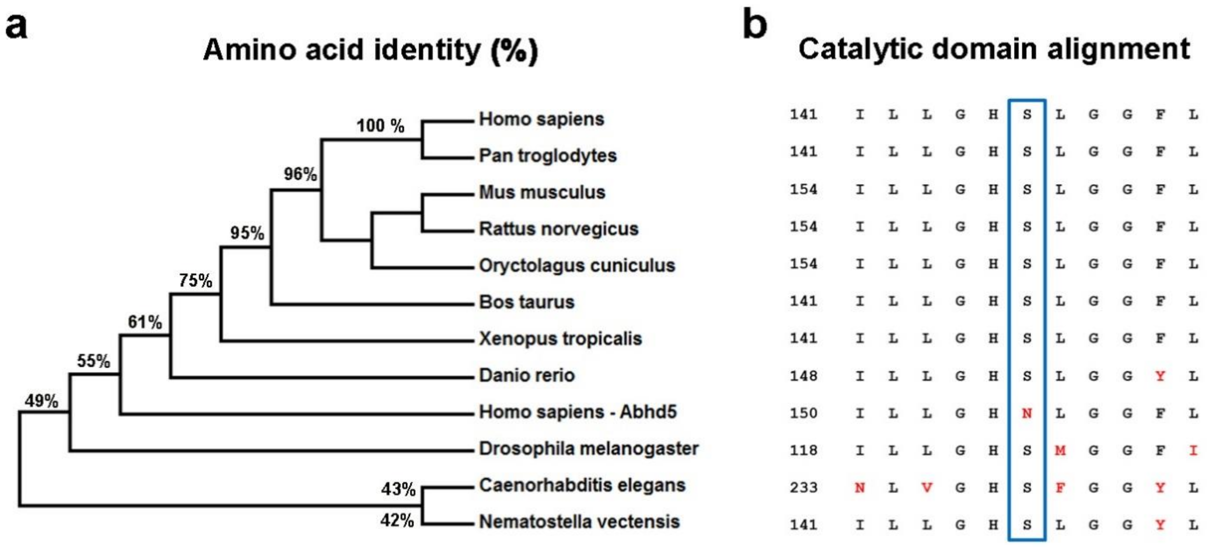

Supplementary Fig. 8. ABHD4 is an evolutionarily conserved serine hydrolase. a The phylogenetic tree of ABHD4 protein sequences. Percentage values indicate the amino acid identity between the human ABHD4 protein sequence and the respective orthologues of other species. Human ABHD5, the closest relative of ABHD4 is also included for comparison. **b** Note that ABHD4 orthologues exhibit substantial homology that is especially high around the catalytic serine residue (blue box) and the consensus nucleophile elbow sequence GXSXG. In contrast, ABHD5, the paralogous enzyme that diverged from ABHD4 ~500 million years ago, lost its catalytic serine during evolution.

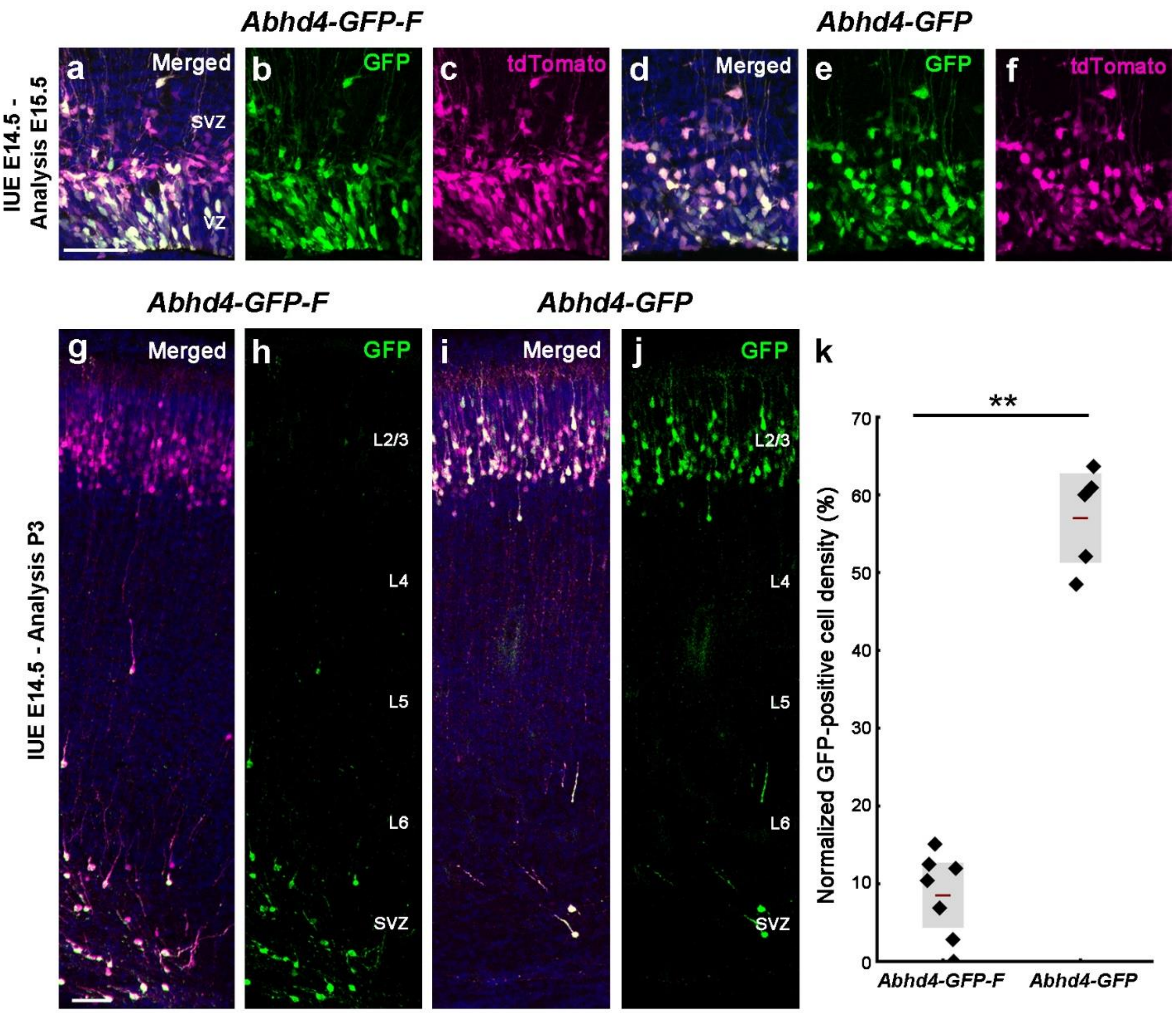

Supplementary Figure 9.

Supplementary Fig. 9. Downregulation of overexpressed ABHD4 is required for delayed migration into the cortical plate. **a-f,** Expression of both fused *Abhd4-GFP* (labeled *Abhd4-GFP-F*; **a-c**) and bicistronic constructs (labeled *Abhd4-GFP*; **d-f**) completely overlaps with the co-electroporated tdTomato 24 hours after electroporation. **g-h,** By postnatal day 3 (P3), some pyramidal cells expressing tdTomato undergo delayed migration and appear in layer 2/3. However, the *GFP*-containing fusion construct is not detectable in the cortical plate indicating the specific downregulation of the ABHD4-GFP fusion protein in the surviving neurons, because those few cells that remain captured in the SVZ still colocalize GFP and tdTomato. In these cells, the GFP tag likely reduces the efficiency of the downregulation of the ABHD4-GFP protein. **i-j,** In contrast, in case of the bicistronic construct from which ABHD4 and GFP translation is independent, GFP colocalizes with tdTomato in surviving cells that migrate into the cortical plate. **k,** Normalized GFP density at P3 is significantly lower in cortical samples electroporated with the fusion vs. the bicistronic construct (two-sided Mann-Whitney U test,***P* = 0.0025; *n* = 5 animals per *Abhd4-GFP* treatment, *n* = 7 animals per *Abhd4-GFP-F* electroporation; data was normalized to tdTomato cell number). Graphs show box-and-whisker plots (including minima, maxima and median, lower and upper quartiles) with values representing averages per animals. IUE, *in utero* electroporation; SVZ, subventricular zone; VZ, ventricular zone; L2/3-L6 mark the cortical layers. Scale bars: **a-f:** 50 µm; **g-j**: 100 µm. Source data are provided as a Source Data file.

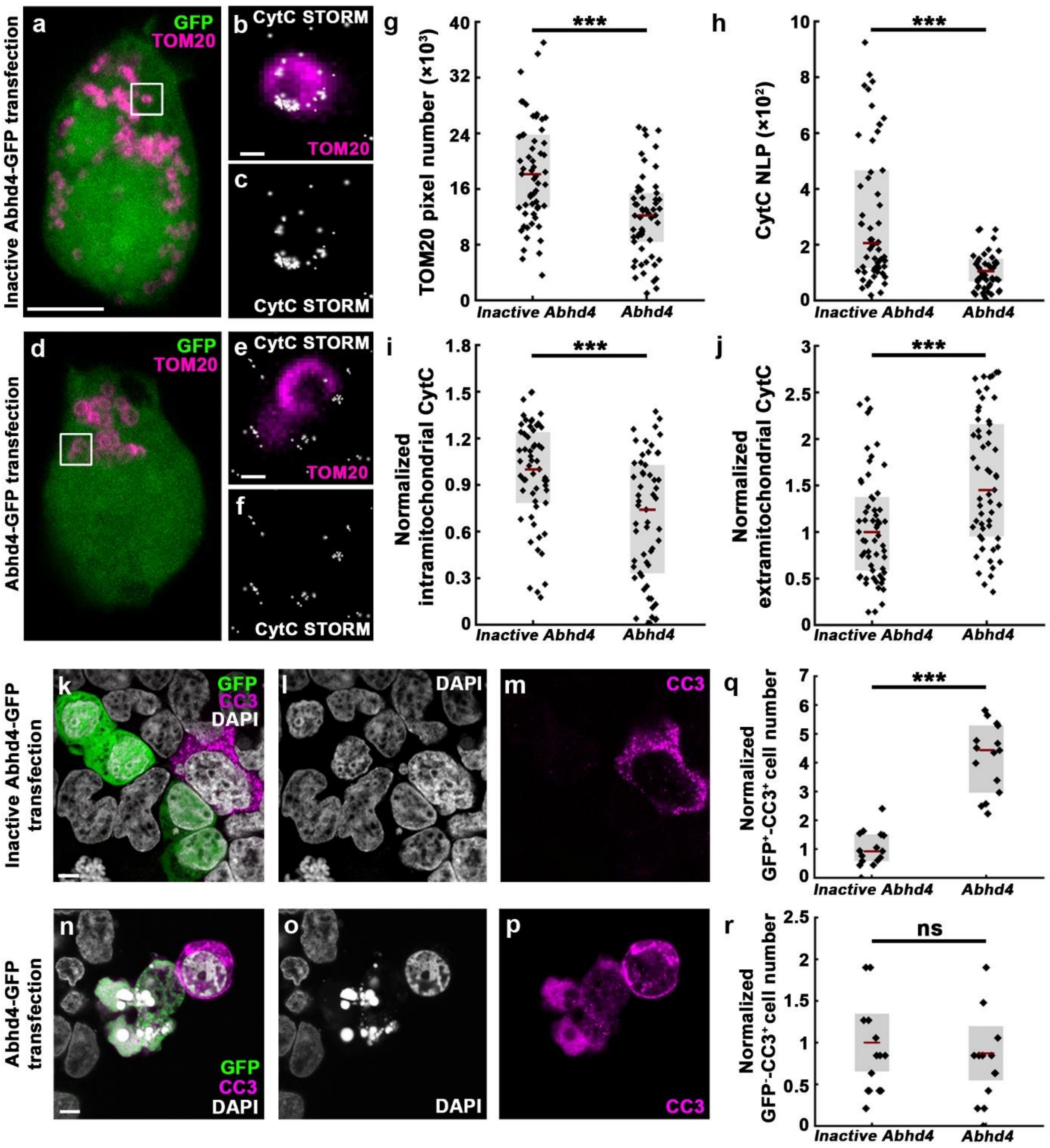

Supplementary Figure 10.

Supplementary Fig. 10. ABHD4 drives apoptosis cell-autonomously. a-f Correlated confocal and STORM super-resolution images of mitochondrial import receptor subunit TOM20 homolog (TOM20)- and cytochrome c (CytC)-immunostaining, respectively, of HEK293 cells transfected either with wild-type (**d**) or with an enzymatically inactive form of *Abhd4-GFP* (**a**). Note that STORM localization points representing individual CytC proteins are concentrated in the TOM20-positive mitochondrion in the transfected HEK293 cell under control condition (**b,c**), but are released into the extramitochondrial environment in the *Abhd4-GFP* electroporated cell (**e,f**). **g** Quantification of TOM20-immunostaining in transfected HEK293 cells. **h** The number of localization points (NLP) representing CytC in the same cells. **i** Normalized number of CytC-localization points within TOM20-positive mitochondria. **j** Normalized number of CytC-localization points outside of TOM20-positive mitochondria. Two-sided Mann-Whitney U test in **g-j**, ***P < 0.0001 in **g,h** ; ***P = 0.0003 in **i,j**; n = 61 cells from n = 4 cultures per Inactive *Abhd4-GFP* treatment, n = 57 cells from n = 4 cultures per *Abhd4-GFP* treatment. **k-p** Confocal images show cleaved caspase-3 (CC3)-immunostaining in HEK293 cells after electroporation with *Abhd4-GFP* (**k-m**), but not with Inactive *Abhd4-GFP* (**n-p**). Besides the molecular evidence, the characteristic apoptotic signs of cell shrinkage and nuclear fragmentation induced by ABHD4 are also notable (**n-p**). **q** Quantification of the ratio of GFP-expressing HEK293 cells that are immunostained for CC3 (two-sided Mann-Whitney U test, ***P < 0.0001; n = 15 cells from n = 3 cultures per both treatments). **r** Statistical analysis of GPF-negative, but cleaved caspase-3-positive normalized cell number (two-sided Student's unpaired t-test, P = 0.5972). Graphs show box-and-whisker plots (including minima, maxima and median, lower and upper quartiles, except (**r**) where mean values with 2 × standard error are presented) with single values. Scale bars: **a,d, k-p:** 5 µm; **b, c, e, f**: 500 nm. Source data are provided as a Source Data file.

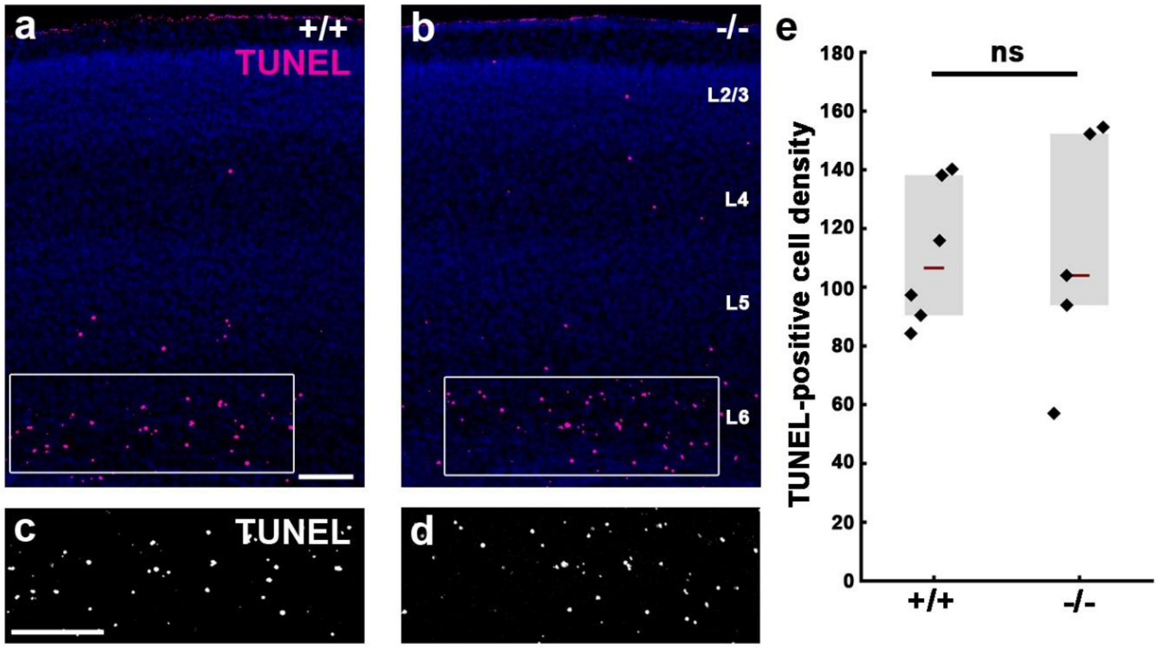

Supplementary Fig. 11 | ABHD4 is not required for developmentally controlled programmed cell death in the developing neocortex. a-d, Comparable levels of programmed cell death in the early postnatal day 3 (P3) cerebral cortex of wild-type (+/+) and *Abhd4*-knockout (-/-) mice. **e,** Quantification of the density of TUNEL-positive dead cells (two-sided Mann-Whitney U test, $P = 0.792$; from $n = 6$ animals per wild-type (+/+) mice, $n = 5$ animals per *Abhd4*-knockout (-/-) mice). L2/3-6 mark the cortical layers. Data are shown as box-and-whisker plots (including minima, maxima and median, lower and upper quartiles) with values representing averages per animals. Scale bars: **a-d:** 100 µm. Source data are provided as a Source Data file.

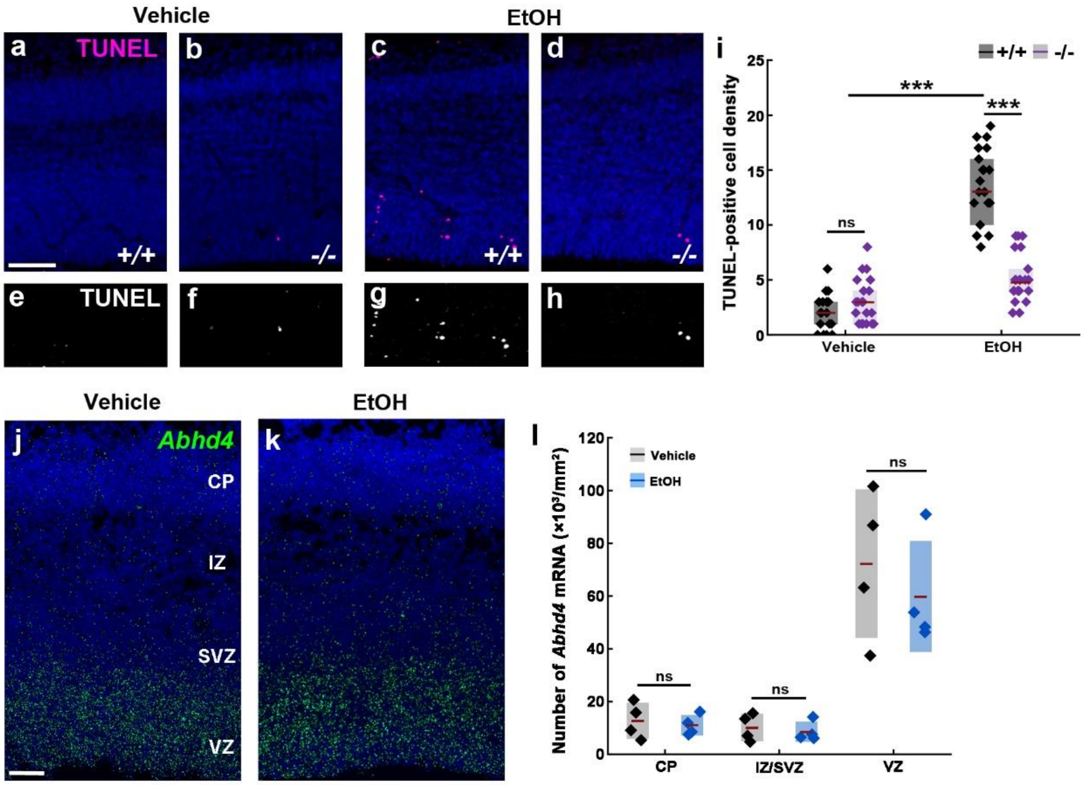

Supplementary Fig. 12 | *Abhd4* absence protects the prenatal cortex from cell loss caused by acute ethanol treatment. a-h A single maternal exposure to alcohol evokes cell death in the developing neocortex of wild-type (**c,g**), but not in *Abhd4*-knockout embryos (**d,h**). **i** Quantification of cell death in the acute alcohol model (Kruskal-Wallis test with post hoc Dunn's test, ***$P < 0.0001$; ns = not significant, $P \approx 1$; $n = 18$ sections from $n = 3$ animals per wild-type, vehicle-treated mice, $n = 21$ sections from $n = 3$ animals per *Abhd4*-knockout, vehicle-treated mice, $n = 22$ sections from $n = 3$ animals per wild-type, ethanol-treated mice, $n = 23$ sections from $n = 3$ animals per *Abhd4*-knockout, ethanol-treated mice). **j-k** Representative images for *Abhd4* fluorescent *in situ* hybridization after acute ethanol treatment (**k**) and vehicle control (**j**). **l** Statistical analysis of *Abhd4* mRNA quantity in three different area corresponding to the VZ, SVZ/IZ and CP in both vehicle- and ethanol-treated cortices (two-sided Mann-Whitney U test, ns = not significant, CP: $P = 0.89$; IZ/SVZ $P = 0.88$; VZ: $P = 0.68$; $n = 4$ animals in all cases). Graphs show box-and-whisker plots (including minima, maxima and median, lower and upper quartiles) with single values (**i**) or with values representing averages per animals (**l**). MZ: marginal zone, CP: cortical plate, IZ: intermediate zone, SVZ: subventricular zone, VZ: ventricular zone. Scale bars: **a-h:** 100 µm; **j-k:** 50 µm. Source data are provided as a Source Data file.

Supplementary Table 1. Primer sequences used for cloning

Gene name	Fragment size (bp)	Forward primer 5'	Reverse primer 3'
Cdh2	896	ATGTGCCGGATAGCGGGAGCGC	TCAGTCGTCACCACCGCCGTACATG
Abhd4	1068	ATGGGCTGGCTCAGCTCGACCCG	TCAGTCAACTGAGTTGCAGATCTCT
Inactive Abhd4	1068	CCGTGCACCTCCAACCTGGGTCAGGGCTGTGGCATCTGTCC	GGACAGATGCCACAGCCCTGACCCAGGTTGGAGGTGCACGG
Short Abhd4	418	CGGCAGGGCTTGTTTACTAT	TCTCCCGCCATGTC TCTATT
Slc1a3	406	TAGGGGCAGGCTGTGTGTGGCTCAC	TCGTTTCTTTGGGGGCTGATTAGGGAC

Supplementary Table 2. Primary antibodies used in the study

Name	Species	Dilution	Company	Cat. number	Lot number
PHH3	rabbit	1:500	Millipore	06-570	1957281
Validation: Evaluated by Western Blot in Colcemid treated HeLa acid extract.<br>Western Blot Analysis: 0.5 – 1 µg/ml of this antibody detected Histone H3 on 10 µg of Colcemid treated HeLa acid extract.					
TBR1	rabbit	1:500	Abcam	ab31940	GR305594-1
Validation: Antibody has been referenced in 250 publications according to the manufacturer's website, including: Hattori et al. 2020; Nature Communications[1]					
TBR2	rabbit	1:500	Abcam	ab23345	GR3179448-1
Validation: Popovitchenko et al 2020; Nature Communications[2]					
TBR2	rat	1:200	Invitrogen	14-4875-82	2135830
Validation: Antibody was referenced in several publications including Wang et al. 2016; Nature Neuroscience[3]					
LAMA1	rabbit	1:500	Sigma	L9393	025M4846V
Validation: Zhang et al 2019; Nature Communications[4]					
PAX6	rabbit	1:300	Biolegend	901301	B201255
Validation: Antibody was referenced in 149 publications according to the manufacturer's website: Quadrato et al. 2017; Nature[5]					
CC3	rabbit	1:500	Cell Signaling	9661S	0043
Validation: Antibody was used I 4774 publication according to the manufacturer's website: https://www.cellsignal.com/products/primary-antibodies/cleaved-caspase-3-asp175-antibody/9661					
Nestin	mouse	1:1000	Millipore	MAB353	2987440

Validation: Antibody was used in 165 publications including Ye et al 2014; Nature Communications[6]					
BrdU	mouse	1:400	Sigma	B8434	118K4835
Validation: Zhang et al. 2014; Carcinogenesis[7]					
TOM20	rabbit	1:1000	Santa Cruz	s-11415	C0614
Validation: Cserép et al. 2020; Science[8]					
CytC	mouse	1:2000	Biolegend	612302	B169559
Validation: Cserép et al. 2018; eNeuro[9]					
GFP	goat	1:1000	Abcam	ab5450	GR277059
Validation: Western blot assay with recombinant GFP protein using as positive control.					
Catalase	rabbit	1:3000	Abcam	ab1877	21101-1
Validation: Western blot assay in an earlier publication[10].					
ABHD4	rabbit	1:500	ImmunoGenes LTD.	na	na
Validation: Western blot assay using Abhd4 -/- animals, further information in the Material and Methods section.					

References:

1. Hattori, Y. *et al.* Transient microglial absence assists postmigratory cortical neurons in proper differentiation. *Nat. Commun.* **11**, 1631 (2020).

2. Popovitchenko, T. *et al.* Translational derepression of Elavl4 isoforms at their alternative 5′ UTRs determines neuronal development. *Nat. Commun.* **11**, 1674 (2020).

3. Wang, L., Hou, S. & Han, Y.-G. Hedgehog signaling promotes basal progenitor expansion and the growth and folding of the neocortex. *Nat. Neurosci.* **19**, 888–896 (2016).

4. Zhang, Z. *et al.* Brain-targeted drug delivery by manipulating protein corona functions. *Nat. Commun.* **10**, 3561 (2019).

5. Quadrato, G. *et al.* Cell diversity and network dynamics in photosensitive human brain organoids. *Nature* **545**, 48–53 (2017).

6. Ye, T., Ip, J. P. K., Fu, A. K. Y. & Ip, N. Y. Cdk5-mediated phosphorylation of RapGEF2 controls neuronal migration in the developing cerebral cortex. *Nat. Commun.* **5**, 4826 (2014).

7. Zhang, H. *et al.* Sohlh2 inhibits ovarian cancer cell proliferation by upregulation of p21 and downregulation of cyclin D1. *Carcinogenesis* **35**, 1863–1871 (2014).

8. Cserép, C. *et al.* Microglia monitor and protect neuronal function through specialized somatic purinergic junctions. *Science.* **367**, 528–537 (2020).

9. Cserép, C., Pósfai, B., Schwarcz, A. D. & Dénes, Á. Mitochondrial ultrastructure is coupled to synaptic performance at axonal release sites. *eNeuro* **5**, (2018).

10. Choi, S. *et al.* Decreased Catalase Expression and Increased Susceptibility to Oxidative Stress in Primary Cultured Corneal Fibroblasts from Patients with Granular Corneal Dystrophy Type II. *Am. J. Pathol.* **175**, 248–261 (2009).
