# Supplementary material for: ABHD4-dependent developmental anoikis safeguards the embryonic brain
Source: Nat Commun. 2020 Aug 31;11:4363. doi: 10.1038/s41467-020-18175-4 (PMC7459116; doi:10.1038/s41467-020-18175-4)
Supplement: Supplementary file 3 — Reporting Summary [file 41467_2020_18175_MOESM3_ESM.pdf]

nature research

Corresponding author(s): István Katona

Last updated by author(s): Jul 27, 2020

Reporting Summary

Nature Research wishes to improve the reproducibility of the work that we publish. This form provides structure for consistency and transparency in reporting. For further information on Nature Research policies, see Authors & Referees and the Editorial Policy Checklist .

Statistics

For all statistical analyses, confirm that the following items are present in the figure legend, table legend, main text, or Methods section.

n/a	Confirmed	
☐	☒	The exact sample size (*n*) for each experimental group/condition, given as a discrete number and unit of measurement
☐	☒	A statement on whether measurements were taken from distinct samples or whether the same sample was measured repeatedly
☐	☒	The statistical test(s) used AND whether they are one- or two-sided<br>*Only common tests should be described solely by name; describe more complex techniques in the Methods section.*
☐	☒	A description of all covariates tested
☐	☒	A description of any assumptions or corrections, such as tests of normality and adjustment for multiple comparisons
☐	☒	A full description of the statistical parameters including central tendency (e.g. means) or other basic estimates (e.g. regression coefficient) AND variation (e.g. standard deviation) or associated estimates of uncertainty (e.g. confidence intervals)
☐	☒	For null hypothesis testing, the test statistic (e.g. *F*, *t*, *r*) with confidence intervals, effect sizes, degrees of freedom and *P* value noted<br>*Give P values as exact values whenever suitable.*
☒	☐	For Bayesian analysis, information on the choice of priors and Markov chain Monte Carlo settings
☒	☐	For hierarchical and complex designs, identification of the appropriate level for tests and full reporting of outcomes
☐	☒	Estimates of effect sizes (e.g. Cohen's *d*, Pearson's *r*), indicating how they were calculated

Our web collection on statistics for biologists contains articles on many of the points above.

Software and code

Policy information about availability of computer code

Data collection	In situ and immunofluorescence images: Eclipse 80i upright microscope equipped with a DS-Fi1 CCD camera (Nikon), A1R confocal laser-scanning system built on a Ti-E inverted microscope and operated by NIS-Elements AR software 4.50.00 (Nikon).<br>STORM superresolution microscopy: CFI Apo TIRF 100× objective (1.49 NA) on a Ti-E inverted microscope equipped with an N-STORM system, a C2 confocal scan head (Nikon), and an iXon Ultra 897 EMCCD camera (Andor). The system was operated by NIS-Elements AR software version 4.40.00 (Nikon).<br>Western blots: PowerPac HC High-Current Power Supply (Bio-Rad).<br>Ethanol serum level determination: Synchron Systems Ethanol assay kit (Beckman Coulter).
Data analysis	The following commercial software and open source codes were used in data analysis: Fiji/ImageJ (2.0), Nikon NIS-Elements AR (5.02), Adobe Photoshop CS5, Stastisca 13.1, Prism5, Phylogenetic tree: Mega7 with Maximum Parsimony method and Subtree-Pruning-Regrafting algorithm (https://www.megasoftware.net/), Convex hull: Visual Molecular Dynamics software (version 1.9.3), VividSTORM (http://katonalab.hu/vividstorm2/), Phyton (2.7). A custom Python (2.7)-based script was written to analyze STORM localizations points in mitochondrial profiles.

For manuscripts utilizing custom algorithms or software that are central to the research but not yet described in published literature, software must be made available to editors/reviewers. We strongly encourage code deposition in a community repository (e.g. GitHub). See the Nature Research guidelines for submitting code & software for further information.

Data

Policy information about availability of data

All manuscripts must include a data availability statement. This statement should provide the following information, where applicable:

- Accession codes, unique identifiers, or web links for publicly available datasets
- A list of figures that have associated raw data
- A description of any restrictions on data availability

The data that support the findings of this study are available from the corresponding author upon reasonable request. Source data are provided with this paper which includes all statistical results regarding Figure 1k, p, 2k, t, 3k, n, 4c, f, o, p, 5d, h, o, 6e, l, m, 7i and Supplementary Figs 1m, n, 2j, q, 3d, 5j, k, 6c, f, i, 7c, f, i, 9k, 10g, h, i, j, q, r, 11e, 12i, l as well as uncropped images of western blots. In silico analysis were made by using two different datasets: GSE38805 (embryonic mouse cortex) [https://www.ncbi.nlm.nih.gov/geo/query/acc.cgi?acc=GSE38805] and GSE75140 (fetal human cortex and cerebral organoid) [https://www.ncbi.nlm.nih.gov/geo/query/acc.cgi?acc=GSE75140].

Field-specific reporting

Please select the one below that is the best fit for your research. If you are not sure, read the appropriate sections before making your selection.

☒ Life sciences ☐ Behavioural & social sciences ☐ Ecological, evolutionary & environmental sciences

For a reference copy of the document with all sections, see nature.com/documents/nr-reporting-summary-flat.pdf

Life sciences study design

All studies must disclose on these points even when the disclosure is negative.

Sample size	Sample size was predetermined based on literature data using the same well established experimental approaches in identical animal models in our laboratory (Cserép et al., Science 367, 528–537 (2020)).
Data exclusions	Samples were only excluded from the data analysis when electroporation was unsuccessful.
Replication	We confirm that all experiments in this study were replicated successfully at least three times in three different mice or in three independent cell cultures. The same experimental protocol was followed by identical steps of data analysis. Source Data contains exact number of samples and animals used in this study.
Randomization	All animals and cell culture wells were selected randomly.
Blinding	The experimenter was always blind to group allocation such as mouse genotype or treatment type. In some cases of electroporation experiments the post hoc analysis were not possible in a completely blinded manner to the striking biological differences between the genotypes (e.g. lack of cell migration).

Reporting for specific materials, systems and methods

We require information from authors about some types of materials, experimental systems and methods used in many studies. Here, indicate whether each material, system or method listed is relevant to your study. If you are not sure if a list item applies to your research, read the appropriate section before selecting a response.

Materials & experimental systems

n/a	Involved in the study	
☐	☒	Antibodies
☐	☒	Eukaryotic cell lines
☒	☐	Palaeontology
☐	☒	Animals and other organisms
☒	☐	Human research participants
☒	☐	Clinical data

Methods

n/a	Involved in the study	
☒	☐	ChIP-seq
☒	☐	Flow cytometry
☒	☐	MRI-based neuroimaging

Antibodies

Antibodies used

The following antibodies were used in this study and validated by the manufacturer and published data:
Rabbit anti-phospho-histone H3 (PHH3, 1:500, Millipore, 06-570, Lot:1957281), rabbit anti-transcription factor T-box, brain, 1 (TBR1 , 1:500, Abcam, ab31940, Lot: GR305594-1), rabbit anti-T-box, brain, 2 (TBR2, 1:500, Abcam, ab23345, Lot:GR3179448-1), rat anti-T-box, brain, 2 (TBR2, 1:200, Invitrogen, 14-4875-82, Lot:2135830), rabbit anti-laminin (LAMA1, 1:500, Sigma, L9393, Lot:025M4846V), rabbit anti-paired box protein-6 (PAX6, 1:300, Biolegend, 901301, clone: poly19013, Lot:B201255), rabbit anti-cleaved-caspase 3 (CC3; 1:500; Cell Signaling; 9661S, Lot:0043), mouse anti-nestin (1:1000, Millipore, MAB353, Lot:2987440),

mouse anti-bromodeoxyuridine (BrdU; 1:400; Sigma; B8434, Lot:118K4835), rabbit anti-TOM20 (1:1000; Santa Cruz; s-11415, Lot:C0614), mouse anti-cytochrome C (CytC; 1:2000; Biolegend; 612302, Clone:6H2.B4, Lot: B169559), goat anti-GFP (1:1000; Abcam; ab5450; Lot: GR277059) rabbit anti-catalase (1:3000; Abcam; ab1877; Lot: 21101-1).

Newly generated antibody:
The specificity of rabbit anti-ABHD4 antibody (1:500; ImmunoGenes Ltd.) was validated by using western blots and Abhd4-knockout control animals.

The following secondary antibodies were used in this study:
Donkey Anti-Rabbit IgG (H+L) Alexa 488 (1:400,Jackson, 711-545-152), Donkey Anti-Rabbit IgG (H+L) Alexa 594 (1:400, Jackson, 711-585-152), Donkey Anti-Mouse IgG (H+L) Alexa 488 (1:400, Jackson, 715-545-150), Donkey Anti-Mouse Alexa IgG (H+L) 647 (1:400, Jackson, 715-605-150), Donkey Anti-Goat Alexa IgG (H+L) 488 (1:400, Jackson, 705-545-147), Donkey Anti- Rabbit Biotium 20098-1 CF-568 (1:1000, Biotium, 20098-1), DAPI (1:2000, Millipore, 508741, Lot:2787312) , Anti Rabbit IGG, HRP-linked antibody (1:3000, Cell signaling, 7074, Lot:0025), Alexa Fluor™ 568 Phalloidin (1:500, Thermo Fisher Scientific, A12380, Lot:1881993).

Validation

The distribution of immunolabeled cells and subcellular profiles observed in the present manuscript corroborated previously published results in case of all commercial antibodies, more information about the validation in the Supplementary Information Table 2.
The custom-made antibody against ABHD4 was validated by western blot using Abhd4-knockout animals, further information in the Material and Methods section.

Eukaryotic cell lines

Policy information about cell lines

Cell line source(s)

HEK293 were a kind gift from Dr. Balázs Gereben, Institute of Experimental Medicine Hungarian Academy of Sciences (Egri et al.; Endocrinology 6, 2356-66 (2016)).

Authentication

The cell line was not authenticated in the authors laboratory.

Mycoplasma contamination

The HEK293 cell line was tested for mycoplasma contamination using PCR in the authors laboratory and all results were negative.

Commonly misidentified lines
(See ICLAC register)

No misidentified lines were used in this study.

Animals and other organisms

Policy information about studies involving animals; ARRIVE guidelines recommended for reporting animal research

Laboratory animals

CD-1 mice were used in standard in utero electroporation experiments. Mice bearing a disruption in the Abhd4 gene was generated from the 129S6/SvEvTac strain and were backcrossed into the C57BL/6 background for more than 10 generations prior to present experiments. Both male and female embryos (E14.5-E18.5) and early postnatal animals (P1;P3;P10) were used in this study. Adult (P60-80) male Abhd4 +/+ and -/- animals were used for in situ hybridization. All mice were hosted under specific-pathogen-free conditions and were maintained at 22-24 °C temperature with 30-50% humidity, 12 hour light/12 hour dark cycle was used at the Medical Genetics Unit of IEM.

Wild animals

The study did not involve wild animals.

Field-collected samples

The study did not involve samples collected from the field.

Ethics oversight

All experiments were approved by the Hungarian Committee of the Scientific Ethics of Animal Research (license numbers: XIV-1-001/2332-4/2012 and PE/EA/354-5/2018), and were carried out according to the Hungarian Act of Animal Care and Experimentation (1998, XXVIII, Section 243/1998), in accordance with the European Communities Council Directive of 24 November 1986 (86-609-EEC; Section 243/1998). The antibody against ABHD4 was generated with permissions 22.1/601/000/2009 and XIV-I-001/2086-4/2012 issued by the Food Chain Safety and Animal Health Directorate of the Government Office of Pest County, Hungary.

Note that full information on the approval of the study protocol must also be provided in the manuscript.
